# Reversible Assembly of Terpyridine Incorporated Norbornene-Based Polymer via a Ring-Opening Metathesis Polymerization and Its Self-Healing Property

**DOI:** 10.3390/polym10101173

**Published:** 2018-10-22

**Authors:** Jookyeong Lee, Hwi Hyun Moon, Keewook Paeng, Changsik Song

**Affiliations:** Department of Chemistry, Sungkyunkwan University, 2066 Seobu-ro, Jangan-gu, Suwon-si, Gyeonggi-do 16419, Korea; jookyung7@naver.com (J.L.); ansgnlgus@naver.com (H.H.M.)

**Keywords:** ring opening metathesis polymerization (ROMP), terpyridine, metal–ligand interaction, self-assembly, self-healing, sol-gel system

## Abstract

We induced a terpyridine moiety into a norbornene-based polymer to demonstrate its self-healing property, without an external stimulus, such as light, heat, or healing agent, using metal–ligand interactions. We synthesized terpyridine incorporated norbornene-based polymers using a ring-opening metathesis polymerization. The sol state of diluted polymer solutions was converted into supramolecular assembled gels, through the addition of transition metal ions (Ni^2+^, Co^2+^, Fe^2+^, and Zn^2+^). In particular, a supramolecular complex gel with Zn^2+^, which is a metal with a lower binding affinity, demonstrated fast self-healing properties, without any additional external stimuli, and its mechanical properties were completely recovered.

## 1. Introduction

Self-healing materials are under intense development and have been recently studied by many researchers as a new and improved route toward the safety, an increased lifetime, better energy efficiency, and improved environmental impact of manmade materials [1,2,3]. The self-healing process can be efficiently utilized in a wide range of applications, such as biological muscles, tissue engineering, 3D/4D printing, and soft actuators [4,5,6]. Several self-healing studies have been reported for polymer systems, such as the requirement of healing agents (including light or heat) [7] and the use of reversible and irreversible covalent bonds, as well as dynamic noncovalent bonding interactions (such as hydrogen bonds, π-π stacking, ionomers, or metal-ion interactions) [8,9] as the healing motifs [2]. The healing capability of a given material does not depend solely on the bonding characteristics of the molecular structures, but also on the chain dynamics, which govern properties, such as the mechanical or reaction kinetics [8].

Although many self-healing strategies have been studied, certain problems remain regarding how to improve the strength and make long-lasting self-healing materials that can self-repair multiple times, without an additional external stimulus or an incidental performance [7]. Metal–ligand complexes have the potential for dynamic healing design motifs, considering the flexibility of its wide-range of thermodynamic and kinetic parameters, leading to the possibility of developing highly tunable materials [2]. In addition, metal–ligand interactions are less sensitive to moisture than hydrogen bonds, which should be beneficial for practical applications [2]. Several self-healing gels and solid materials have been reported to use reversible metal−ligand interactions, for which the most commonly utilized ligand system involves multidentate nitrogen-based aromatic ligands, such as terpyridine [9]. For example, Pierre et al. reported a bis-terpyridine cyclam (BTC) that can exhibit self-healing features with Ni^2+^ ions forming Ni_2_BTC metallo suprapolymer gels [10]. Bode et al. studied self-healing polymer films through the crosslinking of terpyridine, containing polymethacrylate copolymers with different cadmium salts. The crosslinking with cadmium(II) halogenide (i.e., chloride, bromide, and iodide) presumably forms ionic clusters consisting of metal complexes. The presence of these clusters contributes to the healing of these networks [11].

To prepare functionalized polymers, several methods have been established, such as the ionic and free radical polymerization of vinyl monomers, group transfer polymerization (GTP), and more recently, ring-opening metathesis polymerization (ROMP) [12]. Among these methods, ROMP is an attractive method because it is robust, produces an absolutely linear material, and is amenable to the formation of various copolymers of a controlled architecture [13,14]. Polymers produced using ROMP can have a variety of chelating groups including terpyridine moieties, which can act as an anchor group in the binding of metal complexes with a polymer chain [14]. A member of our group recently synthesized a terpyridine-based norbornene polymer using ROMP, and conducted visible-light induced photocatalytic oxidation of benzylamine into imine, to confirm its applicability as a regenerative and repairable photocatalyst. The metallo photo-active complex polymer, which can bind to a ruthenium(II) complex (Ru(bpy)_2_(bpy–tpy)Cl_2_) through metal ions, was applied to a reversible photo-catalyst [13]. Other researchers have reported a chemodosimeter for chemical warfare agents (CWAs), using norbornene-derived terpyridine materials [15]. Although terpyridine-based norbornene polymers have been reported in the application of photocatalysts [13], chemo-sensors [15], and anion exchange membranes [16], they have yet to be reported in studies on self-healing.

Herein, we conducted experiments using transition metal ions and a terpyridine ligand complex gel to confirm not only its reversible properties but also its self-repair capabilities. More importantly, such gels can self-recover without the use of external stimuli or the delivery of healing agents. This approach demonstrates a promising route toward the design of strong, ideal, and self-healing materials that can repeatedly self-repair without degradation or the need for external stimuli.

## 2. Experimental

### 2.1. Materials and Instruments

Cis-5-norbornene-*exo*-2,3-dicarboxylic anhydride, 3-aminopropionic acid and *N,N*-Diisopropylethylamine (DIEA) were purchased from Sigma-Aldrich-Korea (Yongin, Korea). 3-aminopropionic acid and 1-[bis(dimethylamino)methylene]-1*H*-1,2,3-triazolo[4,5-b]pyridinium 3-oxide hexafluorophosphate (HATU) were received from Tokyo Chemical Industry Co., Ltd.-Korea (Seoul, Korea) and Chempep, Inc. (Wellington, FL, USA). UV–vis spectra were obtained using a UV-1800 ENG 240 V SOFT spectrometer (SHIMADZU, Kyoto, Japan). FTIR spectra were recorded using a spectrometer Vertex70 (Bruker Optic, Billerica, MA, USA), equipped with a diamond ATR unit. The H NMR spectra were obtained using a Bruker 500 MHz spectrometer (Bruker, Billerica, MA, USA). The chemical shifts were reported in ppm (δ) with TMS as an internal standard, and the coupling constants (J) were expressed in Hz. Using Agilent Technology 1260 Infinity equipment (Agilent, Santa Clara, CA, USA), the average molecular weight and polydispersity index (PDI) of the polymers were determined through gel permeation chromatography (GPC) with THF as the eluent and polystyrene as the standard. The rheological properties of the metallo-gel were tested using a ARES-G2 rheometer (TA Instruments, New Castle, DE, USA). The gel was swelled in DMF for 10 min to mimic the conditions under self-healing and the sample was placed between parallel plates of 8 mm in diameter. The test was conducted under a fixed strain of 20%, and the frequency was swept from 0.1 to 10 Hz at 28 °C.

### 2.2. Synthesis of Monomer, Polymers, and Metal-Complex Gels

#### 2.2.1. Synthesis of 3-(1,3-dioxo-3′,4,7,7′-tetrahydro-1H-4,7-methanoisoindol-2(3H)-yl)propanoic acid (Compound **1**)

A synthesis was conducted according to the procedure described in Reference [13]. Cis-5-norbornene-*exo*-2,3-dicarboxylic anhydride (0.50 g, 3.0 mmol), 3-aminopropionic acid (0.34 g, 3.8 mmol), and triethylamine (0.039 g, 0.38 mmol) were mixed in 10 mL toluene, in a 25 mL round-bottom flask, fitted using a Dean–Stark apparatus. After refluxing the reaction for 12 h, the reaction mixture was cooled and concentrated using a rotary evaporator. The resulting crude solid was then dissolved in chloroform (15 mL) and washed with acidic water (pH = 1, type of acid) (3 × 30 mL). A white solid was obtained with a yield of 75% (0.54 g). ^1^H NMR (500 MHz, CDCl_3_) δ (ppm): 12.44 (s, 1H), 6.36 (s, 2H), 3.63 (t, *J* = 7.5 Hz, 2H), 3.14 (s, 2H), 2.73 (s, 2H), 2.53 (t, *J* = 7.5, 2H), 1.39 (d, *J* = 10 Hz, 1H), 1.27 (d, *J* = 10 Hz, 1H).

#### 2.2.2. Synthesis of 3-(4-([2,2′:6′,2′’-terpyridin]-4′-yl)phenoxy)propan-1-amine (Compound **2**)

As described elsewhere [13], 3-amino-1-propanol (0.69 g, 9.2 mmol) and KOH (0.69 g, 12 mmol) were mixed in DMSO and stirred for 1 h, at 60 °C. Compound 1 was added to the DMSO solution and stirred for 16 h, at 60 °C. The mixture was poured into ethyl acetate (25 mL) and washed with H_2_O (3 × 50 mL). The organic layer was dried over anhydrous Na_2_SO_4_, filtered, and evaporated, affording a yellow solid product with an 87% (1.0 g) yield. ^1^ H NMR (500 MHz, CDCl_3_) δ (ppm): d 8.71 (d, *J* = 5 Hz, 2H), 8.65–8.62 (m, 4H), 7.87–7.79 (m, 4H), 7.34 (t, *J* = 5 Hz, 2H), 7.04 (d, *J* = 9 Hz, 2H), 4.17 (t, *J* = 6 Hz, 2H), 3.20 (t, *J* = 7 Hz, 2H), 2.22 (m, 2H).

#### 2.2.3. Synthesis of *N*-(3-([2,2′:6′,2″-terpyridin]-4′-yloxy)propyl)-3-(1,3-dioxo-3′,4,7,7′-tetrahydro-1H-4,7-methanoisoindol-2(3H)-yl)propanamide (M1) 

Compound **1** (0.17 g, 0.74 mmol) and 1-[bis(dimethylamino)methylene]-1*H*-1,2,3-triazolo[4,5-b]pyridinium 3-oxide hexafluorophosphate (HATU) [13] (0.34 g, 0.88 mmol) were mixed in DMF (15 mL), in a 25 mL two-neck round-bottom flask, and the mixture was stirred for 20 min in the dark. Compound **2** (0.25 g, 0.81 mmol) was then added to the mixture with continued stirring for another 20 min, at room temperature, in the dark. *N*,*N*-Diisopropylethylamine (DIEA) (0.12 g, 0.88 mmol) was added to the reaction mixture, which was then stirred for 19 h, at room temperature. The mixture was poured into ethyl acetate (25 mL) and washed with an aqueous NaHCO_3_ solution (pH = 9–10) (3 × 50 mL). The organic layer was dried over anhydrous Na_2_SO_4_, filtered, and evaporated, affording a yellow solid product with a 52% (0.21 g) yield. ^1^H NMR (500 MHz, CDCl_3_) δ (ppm): d 8.74 (d, *J* = 5 Hz, 2H), 8.66–8.70 (m, 4H), 7.87–7.79 (m, 4H), 7.36 (t, *J* = 5 Hz, 2H), 7.04 (d, *J* = 9 Hz, 2H), 6.26 (s, 2H), 6.04 (s, 1H), 4.11 (m, 2H), 3.81 (m, 2H), 3.48 (m, 2H), 3.27 (s, 2H), 2.68 (s, 2H), 2.55 (t, *J* = 6 Hz, 2H), 2.05 (m, 2H), 1.52 (d, *J* = 7 Hz, 1H), 1.24–1.26 (m, 1H).

#### 2.2.4. Synthesis of 2-(2-ethylhexyl)-3′,4,7,7′-tetrahydro-1H-4,7-methanoisoindole-1,3(2H)-dione (M2) 

Using the same procedure as in the synthesis of Compound **1**, M2 was synthesized using cis-5-Norbornene-*exo*-2,3-dicarboxylic anhydride (0.50 g, 3.0 mmol), 2-ethyl-1-hexylamine (0.39 g, 3.0 mmol), and triethylamine (0.042 mL, 0.30 mmol). A colorless oil product with a yield of 89% (0.78 g) was obtained. ^1^H NMR (500 MHz, CDCl_3_) δ (ppm): 6.29 (s, 2H), 3.37 (t, *J* = 7 Hz, 2H), 3.27 (s, 2H), 2.67 (s, 2H), 1.75–1.70 (m, 1H), 1.51 (d, *J* = 10 Hz, 1H), 1.31–1.21 (m, 9H), 0.91–0.84 (m, 6H). 

#### 2.2.5. Synthesis of Terpyridine-Functionalized Linear Polymer (P0, P30, P50) 

The different ratios of M1 and M2 were dissolved in a degassed mixture of THF and DMF (5 mL), under a N_2_ atmosphere. A total of 5 mol% of a Grubbs’ second-generation catalyst, which was dissolved in 0.3 mL of THF, was added to the mixture using a syringe, and the mixture was stirred at room temperature for 1 h. The reaction mixture was quenched with butyl vinyl ether, and the product was obtained through precipitation, using MeOH. All synthesized polymers were characterization using ^1^H-NMR and GPC (polystyrene standard) for *M*_n_, *M*_w_, and PDI.

## 3. Results and Discussion

**Scheme 1:**
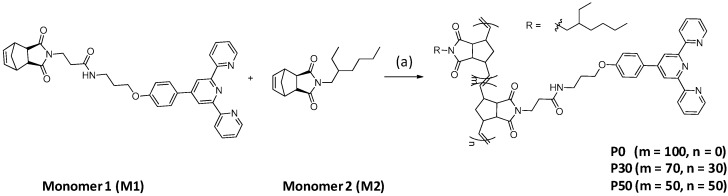
Synthesis of monomers and norbornene-based polymers. Reagents and conditions: (a) Different ratios of M1, M2, and 5 mol% Grubbs’ second-generation catalyst, THF, for 1 h.

To realize self-healing properties, a polymer should possess a reversible binding ability, such as an assembly. As terpyridine is known to be a metal-binding ligand enabling a reversible assembly [11], we incorporated terpyridine groups into the polymer to investigate its self-healing behavior. To prepare the terpyridine-functionalized polymers, norbornene derivatives were chosen as monomers. Norbornene is widely utilized for functional group modifications, and can be easily polymerized through a ring-opening metathesis polymerization (ROMP), using Grubbs’ catalysts [17]. In this study, we utilized Grubbs’ second-generation catalyst to produce a random linear copolymer. The synthetic approach for the norbornene-derived monomers is described in Scheme 1. First, terpyridine- (M1), and ethylhexyl-functionalized norbornenes (M2) were synthesized, starting from *cis*-5-norbornene-*exo*-2,3-dicarboxylic anhydride, through a condensation reaction and an amide coupling reaction, as reported elsewhere [13]. Subsequently, the linear random copolymers P0, P30, and P50 were synthesized with different ratios of M1, M2, using Grubbs’ second-generation catalyst, at room temperature, for 1 h, with ROMP (Table 1). Grubbs’ second-generation catalyst had a sufficiently high activity in ROMP, while providing polymers with slightly larger PDIs than Grubbs’ third-generation catalyst, owing to the slower initiation step [18]. As a result, the terpyridine-functionalized polymers of P30 and P50 were easily synthesized, using ROMP, with terpyridine contents of 30 and 50 mol%, respectively. For comparison, we prepared polynorbornene without a terpyridine moiety (P0), only from M2. Interestingly, it was found that the molecular weight of the polynorbornenes decreased as the mole ratio of terpyridine increased. We suspected that the active Ru catalyst might bind with the terpyridine moiety, during ROMP polymerization, becoming inactive. The higher terpyridine-containing polymer has a higher probability of a Ru complex with terpyridine. In the UV-vis spectrum of P30, a slight absorption was observed at 483 nm, which could be attributed to the singlet–singlet metal-to-ligand charge transfer (MLCT) band from the Ru-terpyridine complex (Figure S1) [19]. Nevertheless, terpyridine did not completely disturb the synthesis of polynorbornenes, when using ROMP with a Grubbs’ catalyst, despite the negative influence on the molecular weight.

All synthesized terpyridine-functionalized polynorbornenes were confirmed using ^1^H-NMR and FT-IR spectroscopic methods. The ^1^H-NMR analysis was carried out by calculating the ratio of terpyridine peak, corresponding to the newly generated olefin in ^1^H-NMR (Figure S2). Based on the results obtained from the ^1^H-NMR analysis, no significant differences were shown between the feed ratios of terpyridine M1 and ethylhexyl M2 and the analytical composition in the synthesized polynorbornenes, which meant that the two monomers showed a similar activity toward the Ru catalyst, when applying ROMP (Table 1).

To confirm that terpyridine had been indeed functionalized well in P30 and P50, we implemented an FT-IR analysis, for a comparison with P0. All polymers were purified using re-precipitation, several times, to remove any unreacted monomers. In the FT-IR spectra of the synthesized polymers, intensities of 2900 cm^−1^ (C-H stretch in aromatics), 1470 cm^−1^ (C-H stretch in aromatic rings), and 1126 cm^−1^ (C-N stretch in amine), and overtone peaks, which correspond to the signature peaks of the terpyridine moiety, increased with respect to the 1700 cm^−1^ C=O peak owing to the increasing proportion of terpyridine moiety, in the polymers, from 0.55 (P30) to 4.01 (P50) (Figure S3). According to the above results, it could be confirmed that the terpyridine moiety was successfully incorporated into the polynorbornenes, when using ROMP.

Interestingly, we found that the content of terpyridine in the polynorbornenes affected their glass transition temperatures (*T*_g_). *T*_g_ was measured with heating and cooling rate of 10 K/min and was determined as the midpoint of the transition on the second cooling cycle. As summarized in Table 1, the measured *T*_g_ values of P30 and P50 (86 °C and 103 °C, respectively) were higher than those of P0 (68 °C), and the more terpyridine that was incorporated, the higher the *T*_g_ that was observed (Figure S4). We suspected that the presence of the terpyridine moiety increased the *T*_g_ of the polymers because it caused intermolecular associations (e.g., a π-π interaction), thereby, restricting the torsional mobility of the polymer chains. Enke et al. also reported increased *T*_g_ values of polymers with a terpyridine ligand [20]. To verify the possible existence of the π-π interaction, we investigated the ^1^H-NMR spectra of terpyridine-incorporated polynorbornenes (P30 and P50) and terpyridine monomer (M1). At different concentrations of P30 and P50, we were not able to find the peak-shifting of the terpyridine moiety. However, we found that terpyridine protons in M1 appeared to shift upfield, when incorporated in polynorbornenes (Figure S5). This upfield shifting seems to accord with previous reports on the π-π interaction of terpyridine moieties [21], which may support our hypothesis for the increase of *T*_g_, with increasing amount of terpyridines. 

**Figure 1:**
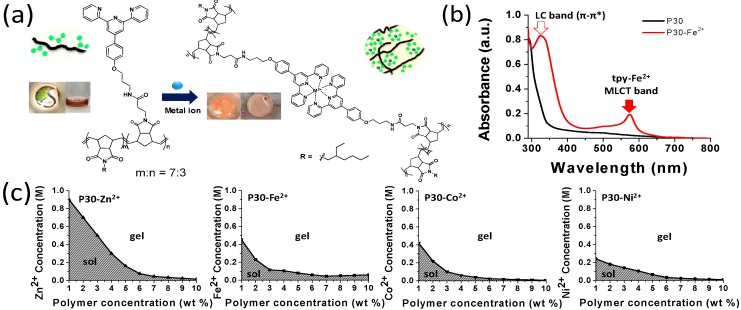
(**a**) Schematic of self-assembly through metal–terpyridine ligand interaction, (**b**) UV-vis spectrum of P30 when adding metal ion Fe^2+^, and (**c**) sol-gel transition graph of P30, depending on different metal ions (Fe^2+^, Ni^2+^, Zn^2+^, and Co^2+^) applied in different polymer concentration solutions (1–10 wt %).

We investigated the sol-gel transition and self-healing properties using terpyridine-functionalized polynorbornenes. When a selected transition metal perchlorate (Fe(ClO_4_)_2_, Co(ClO_4_)_2_, Ni(ClO_4_)_2_, or Zn(ClO_4_)_2_) was added to each polymer solution of P30, gelation occurred because of the terpyridine moiety in the polymer combined with the metal ions (Figure 1a). The UV-vis spectroscopic measurements confirmed that a metal–ligand coordination was indeed formed; ligand planarization and metal-to-ligand charge-transfer (MLCT) bands were observed (Figure 1b). The MLCT bands of the transition metal complexes resulted from the shift in charge density from a molecular orbital with a metal-like characteristic to one with a ligand-like characteristic. In fact, in most cases, a metal-ion induced self-assembly was readily identified, through a visual inspection, owing to the strong coloration of the solution upon metal-ion coordination. For instance, the Fe(II)-terpyridine complex showed a new absorption, at approximately 580 nm, which can be attributed to the MLCT band. As shown in Figure 1b, upon the addition of Fe^2+^, the MLCT band (~580 nm) was clearly observed. In addition, the ligand-centered (LC) band was shifted from ~280 to ~340 nm, owing to the planarization of the terpyridine ligand, upon the binding of Fe^2+^ ions.

To determine the gelation conditions depending on the polymer concentration and type of metal ions, we prepared metal complex gels with varying concentrations of polynorbornene P30 and metal perchlorates (Fe^2+^, Co^2+^, Ni^2+^, and Zn^2+^) (Figure 1c). Each metal ion was continuously added to a given concentration of P30 (e.g., 1 wt % in DMF), up to the point at which the sol began to be converted into a gel. Other polymer solutions of different concentrations (2 to 10 wt %) were subjected to the same procedure described above. As shown in Figure 1c, each gelation point, at which the polymer sol sate was converted into a gel without a flow, was connected, and the shaded areas under the line indicate the sol state of the polymer solution, whereas the area above the line represents the gel state.

When the metal ions Fe^2+^, Ni^2+^, and Co^2+^ were added to a polymer solution, the sol state was rapidly converted into a gel through metal–ligand interactions. In contrast, in the case of the Zn^2+^ ions, it took a relatively long time to obtain a Zn-complex gel, and required a larger amount of zinc ions for the given polymer concentration. We suspect that these trends are related to the metal binding affinity. The generally known binding affinity of metal ions to terpyridine occurs in the order of Fe^2+^ > Ni^2+^ > Co^2+^ > Zn^2+^ [22]. In this study, the Zn^2+^ ions appeared to have the weakest affinity, among the metal-bis-terpyridine complex. We confirmed that the larger the binding constant between terpyridine and the metal ion, the smaller the concentration of the metal ions necessary for the sol-gel transition. In addition, a faster sol-gel transition was observed for the metal ions with a higher binding constant. These observations showed that a sufficient amount of ligands and metal ions are required to form a gel, and the different metal–ligand affinity directly affects the metallogel generation. When we measured the storage moduli (G’) using a rheometer (Figure S6), the G’ of the Fe-complex gel appeared to be higher than that of the Zn-complex gel because of the stronger binding affinity of Fe^2+^ to terpyridine, than to Zn^2+^. However, the stronger binding strength might have reduced the rapid and reversible exchange of metal–ligand interactions, which would have hindered the fast self-healing property. Therefore, we carried out investigations on self-healing by Zn^2+^, since zinc bis-terpyridine complexes exist in dynamic equilibrium with mono-complexes and, thus, exchange rapidly [2,23].

We investigated the self-healing properties of the metallogels and found that the Zn-complex gel of P30 showed a good self-healing behavior through metal–ligand interactions. The higher content of terpyridine in polynorbornene (P50) rendered the metallogels rather compact and less flexible, which was consistent with the higher *T*_g_ of P50 (Table 1). 

The stronger metal–ligand interaction (metallogels with Fe^2+^, Ni^2+^, or Co^2+^) also results in a decrease in the flexibility of the polymer chain and the self-exchange rate of the metal–ligand complex [20]. These results might indicate that, for self-healing, it is very important to ensure the molecular flexibility of polynorbornenes, which could be controlled based on the ratio of terpyridine and the choice of metal ions.

**Figure 2:**
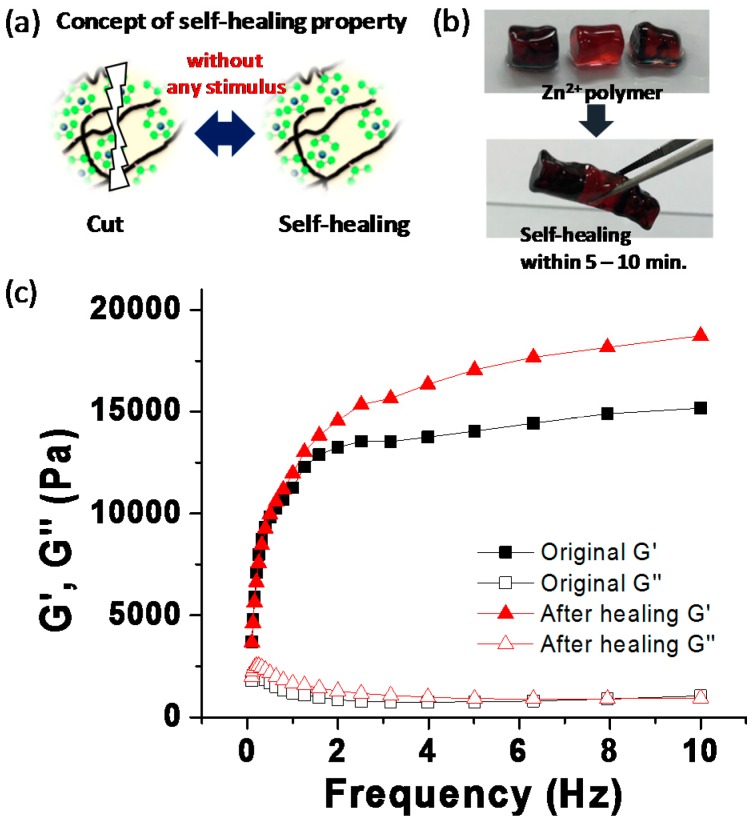
(**a**) Concept of self-healing property with norbornene-based polymer, (**b**) illustration of the self-healing property of the Zn-complex gel, at room temperature, without an external stimulus, and (**c**) storage (G’) and loss (G”) moduli of Zn-complex gel and self-healing gels.

The self-healing performance was tested using a Zn-complex gel, which was cut into three pieces. The middle one was stained with allura red AC, and the other pieces were dyed with disperse blue 3 for easy visualization. When putting the pieces together, the gel was healed completely into a single piece, after 5–10 min at room temperature, without any external stimulus (Figure 2b). To demonstrate the self-healing behaviors of the Zn-complex gel, we conducted rheological tests. The storage (G’) and loss (G’’) moduli of the original Zn-complex gel and the gel, after experiencing the cutting–healing process were measured under a fixed strain of 20%, within a frequency range of 0.1 to 10 Hz. As shown in Figure 2c, the healed gel showed nearly the same values of G’ and G’’ as the original Zn-complex gel, which indicated a good self-healing capacity. These results suggested that the Zn-complex gel from P30 exhibited a rapid recovery in the presence of a dynamic metal–ligand interaction.

In order to compare the effect of counter-ions (anions), we investigated the gelation and self-healing properties from P30 and various Zn salts, with several counter-ions (ClO_4_^−^, CN^−^, Cl^−^, SO_4_^2−^, Br^−^). It should be noticed that all other conditions were exactly same, except for the type of the counter-ion. As shown in Figure S7, the gelation time seemed to be greatly affected by the type of counter-ions, and accordingly, the time needed for self-healing. Although the trend was not completely accordance with the reported binding affinity of anions to Zn^2+^ [24], we confirmed that, in general, strong binding anions retarded the gel formation and self-healing, while weak or no binding species resulted in faster responses. For example, the gelation was instant and the self-healing was the fastest with ClO_4_^−^, the anion of the weakest binding affinity. On the other hand, the stronger binding SO_4_^2−^ showed rather slow gelation and self-healing. Interestingly, we found that the gelation with bromide was not effective and resulted in a very sticky, gum-like solid. We found indeed that the type of anions (counter-ions) significantly affected the gelation and the self-healing property, presumably due to the differences in binding strength to Zn^2+^, size, and polarizability of the anions, although the exact mechanism needs further investigation.

## 4. Conclusions

Self-healable norbornene-based copolymers with terpyridine units of different ratios were synthesized using ROMP. The addition of transition metal ions (Ni^2+^, Co^2+^, Fe^2+^, and Zn^2+^) into the solutions of terpyridine-functionalized polynorbornenes resulted in supramolecularly assembled metallogels, which was demonstrated in detail through UV-vis spectra. Through sol-gel transition experiments, we showed that the content of the terpyridine units and the strength of the metal–terpyridine interaction influence the gelation and self-healing behavior, presumably, owing to the effects on the chain flexibility. In particular, the Zn-complex gel showed a rapid and complete self-healing property without any stimulus, owing to the weak but dynamic metal–ligand interaction.

## Figures and Tables

**Table 1:** Comparison of the feed ratio with the analytical composition of terpyridine-norbornene (M1) and ethylhexyl-norbornene (M2) in synthesized polynorbornenes, and the molecular weights (*M*_n_, and *M*_w_), polydispersity index (PDI), and glass transition temperature (*T*_g_).

Polymer	Feed Ratio (M1:M2)	Analytical Composition * (M1:M2)	*M*_n_	*M*_w_	PDI	*T*_g_ (°C)
P0	0:10	0:10	147,000	254,000	1.7	68
P30	3:7	2.9:7.1	33,100	43,000	1.3	86
P50	5:5	4.1:5.9	19,700	34,100	1.7	103

* Analyzed using ^1^H-NMR.

## Supplementary Materials

The following are available online at http://www.mdpi.com/2073-4360/10/10/1173/s1, Figure S1: UV-vis spectra of P30, Figure S2: 1H-NMR spectra of P0, P30, and P50, Figure S3: FT-IR spectra of M1, P0, P30, and P50, Figure S4: DSC data of P0, P30, and P50, Figure S5. 1H-NMR spectra of M1, P50, Figure S6: Rheology measurements of Fe-complex and Zn-complex gels, Figure S7: Anion effect on gelation and self-healing time with various Zn salts.
