# Supplementary material for: Reversible Assembly of Terpyridine Incorporated Norbornene-Based Polymer via a Ring-Opening Metathesis Polymerization and Its Self-Healing Property
Source: Polymers (Basel). 2018 Oct 22;10(10):1173. doi: 10.3390/polym10101173 (PMC6403875; doi:10.3390/polym10101173)
Supplement: Supplementary file 1 [file polymers-10-01173-s001.pdf]

Supplementary Information for

Reversible assembly of terpyridine incorporated norbornene-based polymer via ring-opening metathesis polymerization and its self-healing property

Jookyeong Lee, Hwi Hyun Moon, Keewook Paeng, and Changsik Song**

Department of Chemistry, Sungkyunkwan University, 2066 Seobu-ro, Jangan-gu, Suwon-si, Gyeonggi-do 16419 Republic of Korea.

E-mail: paeng@skku.edu and songcs@skku.edu

Contents:

- ✓ **Fig S1.** UV-vis spectra of **P30** ---S2
- ✓ **Fig S2.** ^{1}H-NMR spectra of **P0**, **P30**, and **P50** ---------------------------------------S3
- ✓ **Fig S3.** FT-IR spectra of **M1**, **P0**, **P30**, and **P50** -------------------------------------S4
- ✓ **Fig S4.** DSC data of **P0**, **P30**, and **P50** --S5
- ✓ **Fig S5.** ^{1}H-NMR spectra of **M1**, **P50** --S6
- ✓ **Fig S6.** Rheology measurements of Fe-complex and Zn-complex gels ------------S7
- ✓ **Fig S7.** Anion effect on gelation and self-healing time with various Zn salts -----S8

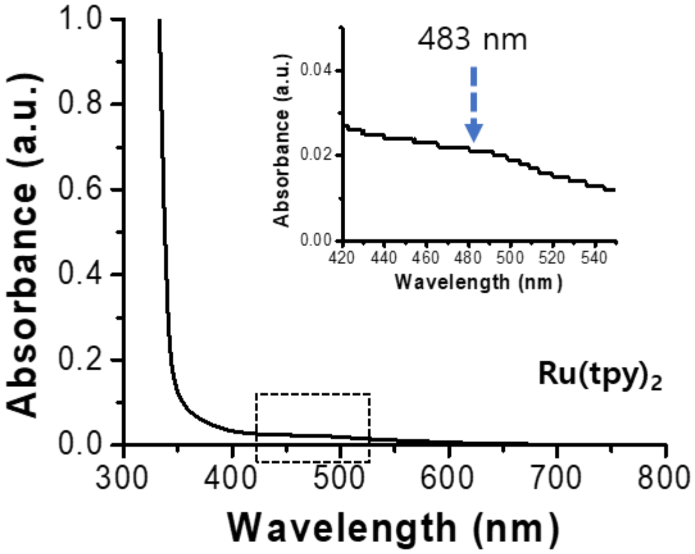

Fig S1. UV−vis absorption spectrum of 0.005 wt% **P30** in DMF. The slight absorbance was observed at a wavelength of 483 nm, which can be attributed to the $Ru(tpy)_2$ complex.

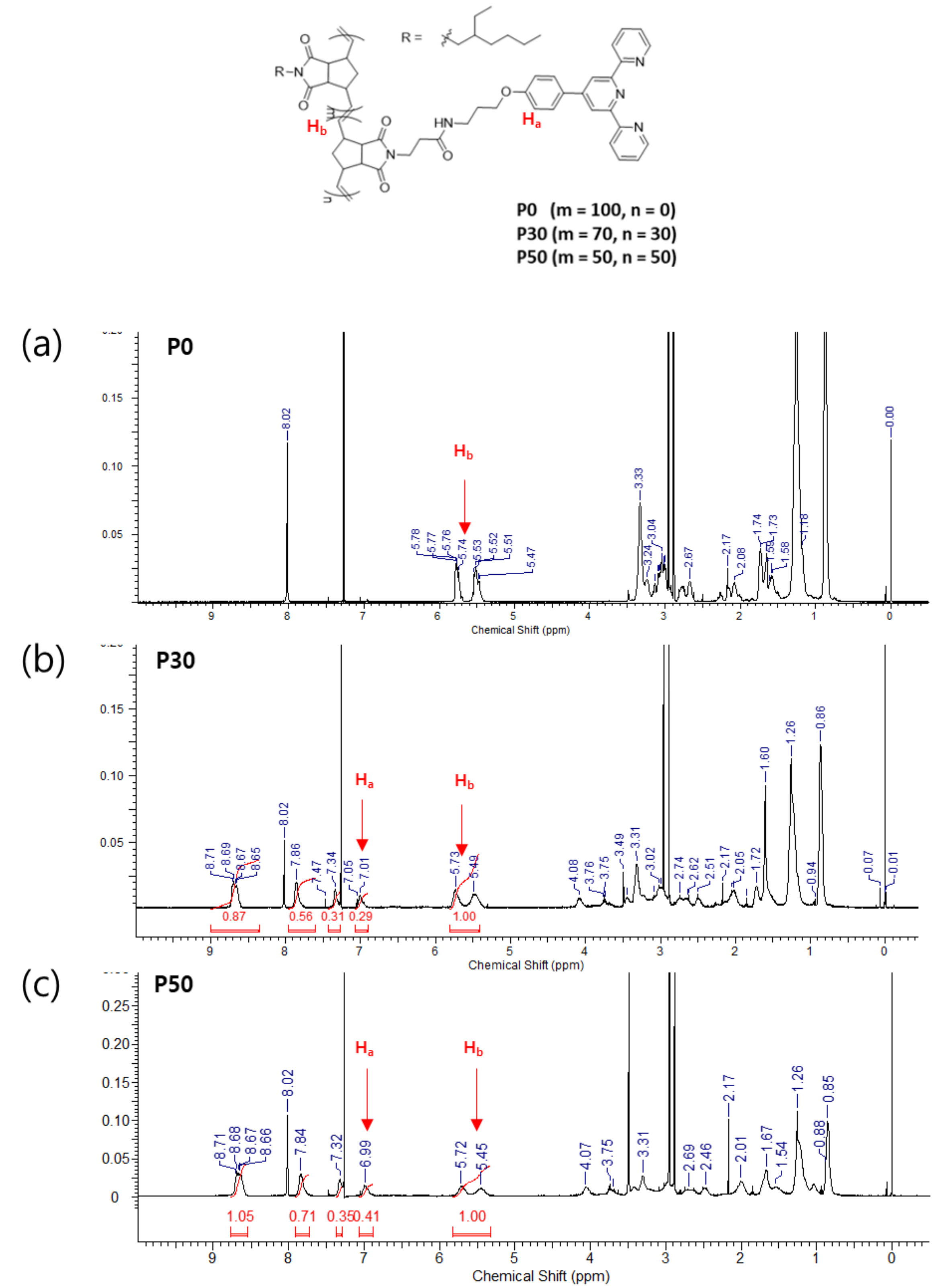

Fig S2. ^{1}H-NMR ($CDCl_3$) spectra of (a) **P0**, (b) **P30**, and (c) **P50**.

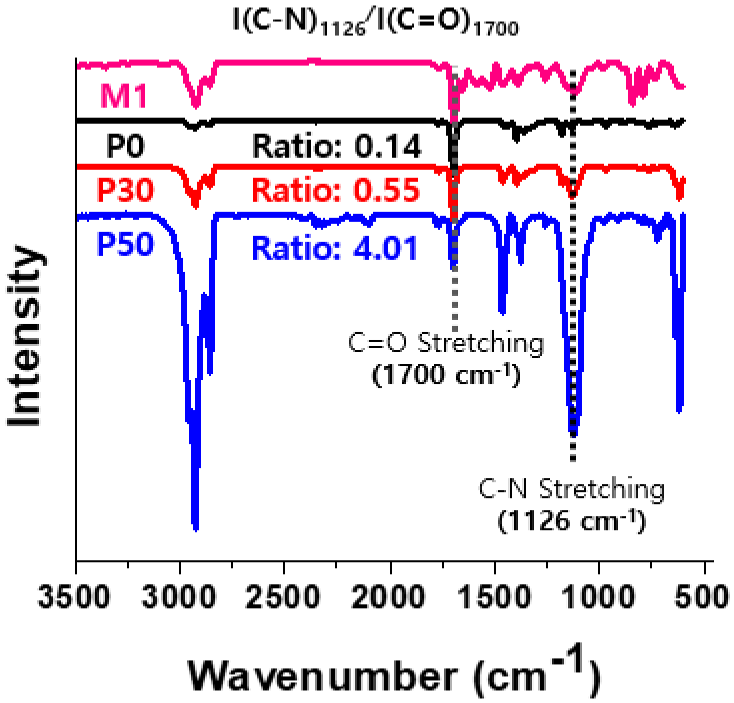

Fig S3. FT-IR analysis of **M1**, **P0**, **P30**, and **P50**. As the ratio of the terpyridine moiety increased, the C-N stretching peak at 1126 cm^{-1} increased.

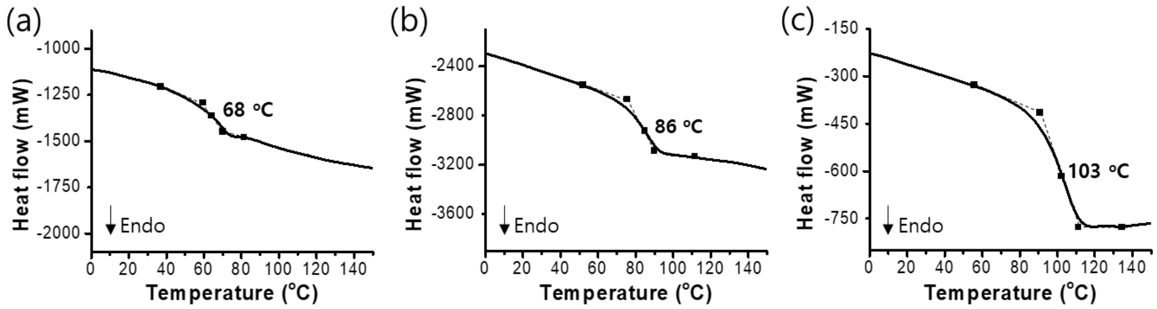

Fig S4. Measurements of glass transition temperature (T_g) by DSC analysis of (a) **P0**, (b) **P30**, and (c) **P50**.

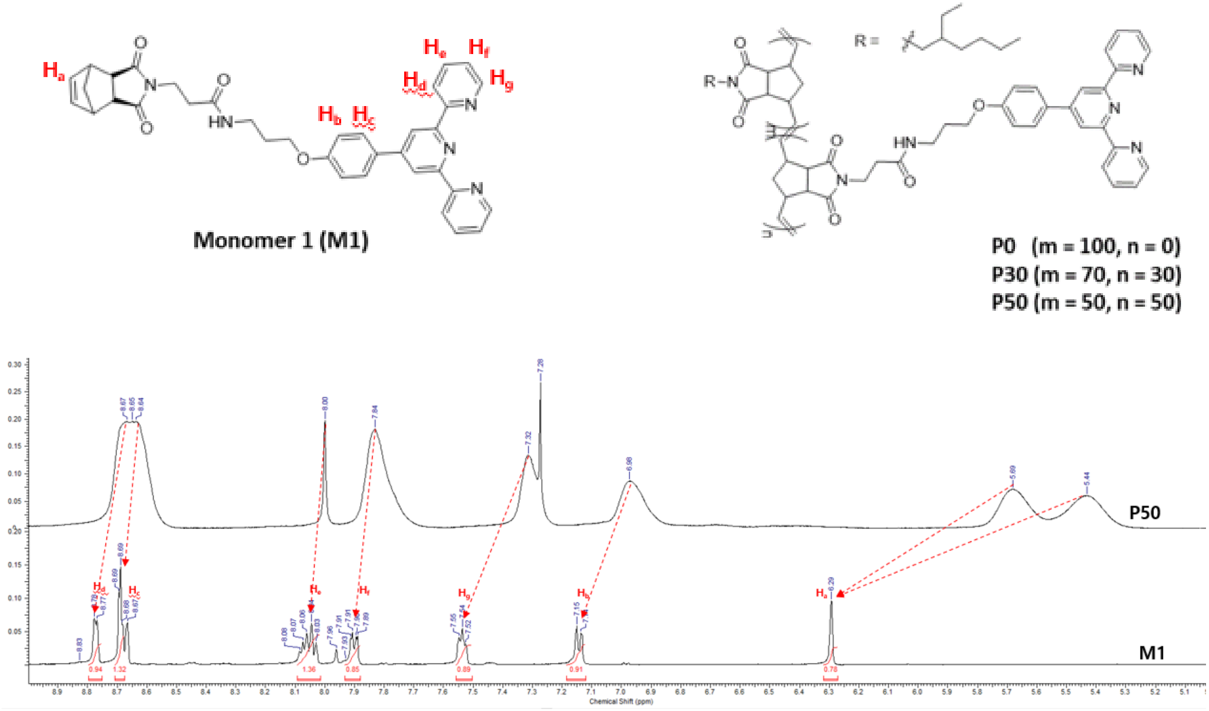

Fig S5. ^{1}H-NMR (DMSO-d_6) spectra of **M1** and **P50**.

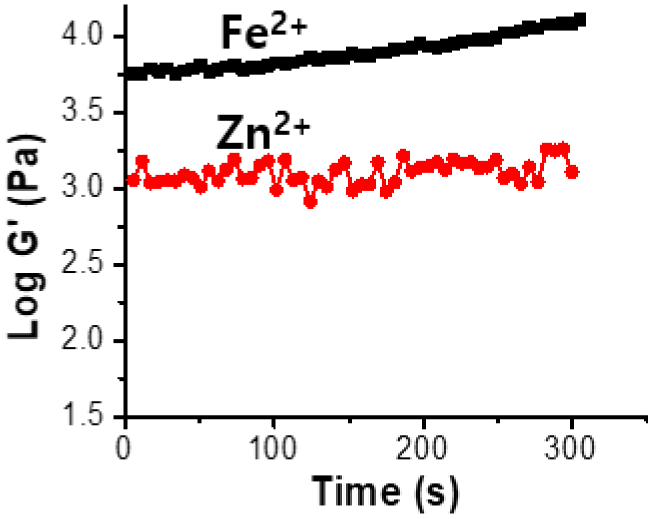

Fig S6. Rheology measurements of Fe-complex and Zn-complex gels.

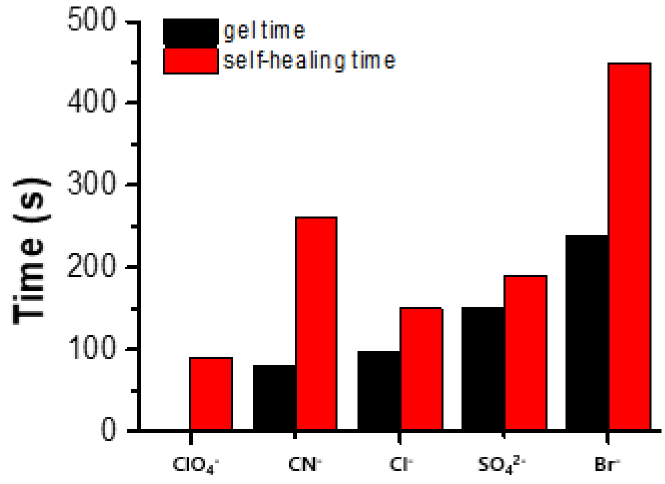

Fig S7. Anion effect on gelation and self-healing time with various Zn salts.
